# Modeling the impact of cochlear nerve degeneration on speech recognition performance

**DOI:** 10.1371/journal.pone.0336299

**Published:** 2025-11-14

**Authors:** Viacheslav Vasilkov, M. Charles Liberman, Stéphane F. Maison

**Affiliations:** 1 Department of Otolaryngology – Head & Neck Surgery, Harvard Medical School, Boston, Massachusetts, United States of America; 2 Eaton-Peabody Laboratories, Mass Eye & Ear, Boston, Massachusetts, United States of America; Universidad de Chile, CHILE

## Abstract

Cochlear nerve degeneration (CND), including the loss of synapses between inner hair cells and auditory nerve fibers (ANFs), has emerged as a likely contributor to “hidden hearing loss”, a condition in which listeners experience speech-in-noise difficulties that cannot be fully explained by audiometric thresholds. This form of primary neural de-afferentation preferentially affects low- and medium-spontaneous rate (SR) fibers, which are critical for encoding acoustic features such as amplitude modulations, especially under challenging listening conditions such as noisy backgrounds. Although CND is well established in animal models and post-mortem human studies, its perceptual consequences remain poorly understood due to the inability to directly assess synaptic integrity in living humans. Here, we combined behavioral testing, a phenomenological model of the auditory periphery, and deep neural network (DNN) decoding to quantify the perceptual impact of SR-specific fiber loss. Audiometric thresholds and word recognition scores for time-compressed, reverberant NU-6 words were obtained from 395 cognitively normal adults aged 18–80. To isolate the neural contribution to speech encoding, we simulated ANF activity under three CND profiles that varied the survival of SR classes, transformed responses into time-frequency neurograms, and decoded them with two DNN architectures trained on word classification. Both networks learned the task, but only the deeper, more constrained model produced recognition scores consistent with human performance and showed sensitivity to CND, with recognition declining as low- and medium-SR fibers were removed. These findings provide a mechanistic link between SR-specific synaptopathy and speech-in-noise difficulties and establish a computational framework for evaluating the perceptual impact of hidden hearing loss.

## Introduction

Understanding speech in noisy environments is a complex perceptual task that becomes more difficult with age, often disproportionately to changes in hearing sensitivity as measured by the audiogram [1,2]. Work in animal models and human autopsy material shows that the loss of sensory cells is often preceded by cochlear nerve degeneration (CND), seen as the loss of synapses between inner hair cells and auditory-nerve fibers (ANFs), followed by degeneration of the axons that connected these synapses to their cell bodies in the spiral ganglion [3–5]. This primary neural loss can occur without elevating behavioral or electrophysiological thresholds until it becomes severe [6,7], largely because the most vulnerable cochlear neurons, those with low spontaneous rates (SRs) and high thresholds, do not contribute to threshold detection in quiet [8,9].

However, these fibers play a critical role in encoding signals in complex acoustic environments. Their higher thresholds and associated response properties support robust coding of suprathreshold sounds when continuous background noise saturates the responses of the more sensitive, high-SR ANFs [10]. Low- and medium-SR fibers extend the dynamic range of hearing, preserve temporal precision when high-SR fibers saturate, and support envelope coding, onset timing, and related cues that are essential for speech intelligibility in noisy or reverberant conditions [11]. These observations motivate the hypothesis that CND may be a key contributor to the perceptual deficits observed in sensorineural hearing loss, particularly speech discrimination difficulties in adverse listening conditions [12].

Consistent with this view, histopathology in human temporal bones links age-related CND to poorer word recognition performance, even after accounting for inner and outer hair cell survival [13]. In aging mice, mRNA expression analysis confirms the selective loss of low-SR fibers [14]. At present, however, SR subgroup identity cannot be determined in human post-mortem tissue, and synaptic integrity at the inner hair cell to ANF junction cannot be assessed *in vivo*. Functional impacts must therefore be inferred indirectly.

Efforts to relate CND to speech-in-noise performance using behavioral, psychophysical, and electrophysiological assays have produced mixed results (see, [15]). Variability across studies likely reflects differences in subject populations, stimulus selection and presentation, outcome measures, and the confounding influence of central auditory and cognitive factors (see [16]). A further complication is that neural loss can be meaningfully interpreted only after accounting for outer hair cell function due to their contributions to ANF sensitivity. Since CND is highly correlated with outer hair cell loss in humans [3,13], statistical adjustment for threshold inevitably entails partial adjustment for neural loss, thereby reducing the power to isolate its perceptual consequences.

Given these limitations, computational models offer a promising approach to isolating the putative effects of CND on speech encoding, but existing approaches differ widely in assumptions and scope. Some models omit SR diversity or use idealized speech materials and lenient decoding strategies, which tends to produce minimal predicted effects of CND (e.g., [17,18]). Others include more physiological and acoustical realistic conditions, yet still report modest decrements that fall short of typical behavioral deficits in aging cohorts (e.g., [19,20]. More recent work that simulates large SR-stratified ANF populations and decodes speech features with neural networks reports stronger impact of synaptopathy, particularly at high degrees of deafferentation [21]. Few of these models have been rigorously validated against human behavioral data, limiting their translational applicability and their potential to inform diagnostic or rehabilitative strategies.

Here, we combine a phenomenological model of the human auditory periphery with deep neural network (DNN) speech decoders trained on spectro-temporal neurograms generated from all three SR fiber populations. We simulate speech stimuli under ecologically relevant conditions, including time compression, reverberation, and a broad range of signal-to-noise ratios, to quantify how SR-specific deafferentation alters suprathreshold speech encoding. This design also allows us to examine how decoder architecture interacts with peripheral neural loss. In parallel, we analyze word-recognition scores from a large cohort of cognitively normal adults tested with time-compressed, reverberant NU-6 words to characterize age-related variability in speech recognition performance and to compare human outcomes with model predictions. Together, we aim to provide a mechanistic framework that links CND to perceptual deficits in aging and generates testable predictions for future experimental and clinical studies.

## Materials and methods

### Participant recruitment and inclusion criteria

We analyzed audiometric thresholds and word-recognition scores from 395 native English-speaking adults (18–80 years). These data were pooled from a series of studies (4/13/2017 – ongoing) that used consistent testing protocols and received approval by the Mass General Brigham Institutional Review Board [16,22–27]. All participants provided written informed consent at the time of their original enrollment. Cognitive screening was performed with the Montreal Cognitive Assessment (MoCA); only individuals with scores ≥ 26 were included. Additional inclusion criteria required good general health and no history of otologic or neurological disorders. At the time of testing, all participants underwent otoscopy and middle-ear screening using the Titan Suite from Interacoustics. Only ears with normal ear-canal volume, tympanic membrane mobility, and middle-ear pressure were included.

### Audiometric threshold and word-recognition testing

Pure-tone air-conduction thresholds were obtained using an Interacoustics Equinox 4.0 audiometer (High Hz option). Standard thresholds were measured from 0.25 to 8 kHz (including 3 and 6 kHz) using TDH-45 headphones. To improve accuracy and reduce variability in the extended high-frequency (EHF) range, thresholds above 8 kHz (9, 10, 11.2, 12.5, 14, and 16 kHz) were measured with warble tones presented via HDA200 circumaural high-frequency headphones.

Speech materials were drawn from the Northwestern University Auditory Test No. 6 (NU-6; Auditec, Inc.), a clinical corpus of 50-item lists of phonemically balanced monosyllabic words [28,29], presented at suprathreshold levels (~40 dB SL; no less than 55 dB HL). Each word follows a consonant-vowel-consonant (CVC) structure and reflect the frequency of phoneme occurrence in American English, ensuring phonetic representativeness and minimizing lexical bias. The absence of semantic or contextual cues makes NU-6 well-suited for assessing suprathreshold speech recognition while limiting top-down influences. NU-6 recordings were sampled at 44.1 kHz; for modeling, stimuli were resampled to 100 kHz before auditory-periphery simulation.

To simulate challenging listening environments and stress peripheral temporal encoding, two signal degradations were applied to each NU-6 words: 1) time compression, which shortens overall duration and reduces cues at phonemic transitions; and 2) reverberation, which spreads temporally and spectrally by convolving each waveform with a synthetic room impulse response with a 0.3-s decay [30]. Specifically, all words were time-compressed to 65% of their original duration using a synchronous overlap-add (SOLA) algorithm and then reverberated with the same impulse response. The same modified NU-6 set was used in human testing and as input to the auditory-nerve model.

### Simulation of auditory-nerve fiber responses to speech stimuli

To quantify how the three SR groups of ANFs contribute to spectro-temporal speech coding, we used a phenomenological model of the human auditory periphery [31]. This model captures key aspects of cochlear signal processing, including nonlinearities associated with basilar membrane filtering, synaptic transmission between inner hair cells and ANFs, and adaptation processes. It also incorporates stochastic properties of synaptic release and accounts partially for the diversity of ANFs by simulating distinct fiber populations with high (100 spikes/s), medium (5 spikes/s), and low (0.1 spikes/s) SRs. To approximate the variability in SRs observed across fibers, a fractional Gaussian noise generator was applied with randomized seeds on each stimulation trial, following prior model implementations [31,32]. All simulations used the *human* parameter set of the Zilany et al. model.

Responses were computed for 71 characteristic frequencies (CFs) logarithmically spaced between 125 Hz and 16 kHz and averaged across 200 repetitions. Outer and inner hair cell functions were set to normal (OHC = 1; IHC = 1) to isolate neural degeneration from pre-neural deficits. Synaptic adaptation was implemented via a cascade of power-law processes, approximated with infinite impulse response filters for computational efficiency [33].

### Correlations between ANF responses and speech

Each time-compressed, reverberated waveform was resampled to 100 kHz, RMS-normalized to ensure consistent intensity scaling, converted to Pascals, and used as model input. The output was a neurogram: a two-dimensional representations of ANF firing rate as a function of post-stimulus time (bin width of 50 μs) and CF (71 CFs logarithmically spaced from 125 Hz and 16 kHz). Neurograms were generated separately for low-, medium-, and high-SR fibers.

To characterize the spectro-temporal features of the speech stimuli and to generate a basis for comparison with simulated neural responses, we applied a continuous wavelet transform (generalized Morse wavelet) to each waveform. This wavelet family offers flexible control over time-frequency tradeoffs and symmetry, making it well suited for analyzing speech dynamics. The real component was half-wave rectified to retain temporal fine structure and envelope fluctuations relevant to phase-locking while suppressing negative-going components that lack physiological interpretability. Wavelet coefficients and ANF responses were both sampled across the same 71 logarithmically spaced frequency channels (125 Hz to 16 kHz).

To quantify the fidelity with which different ANF types captured the spectral and temporal content of the stimulus, we calculated two-dimensional Pearson correlation coefficients between the wavelet spectrograms and the corresponding neurograms using the formula:


$$r=\frac{\sum_{m}\sum_{n}\left(W_{mn}\;-\;\overset{―}{W}\right)\left(v_{mn}\;-\;\overset{―}{v}\right)}{\sqrt{\left(\sum_{m}\sum_{n}{\left(W_{mn}\;-\;\overset{―}{W}\right)}^{\text{2}}\right)\left(\sum_{m}\sum_{n}{\left(v_{mn}\;-\;\overset{―}{v}\right)}^{\text{2}}\right)}}$$

where *W* is two-dimensional matrix of the wavelet coefficient (in Pa) at time index *m* and frequency index *n*, *v* is the ANF firing rate (spikes/s) at the corresponding indices, and $\overset{―}{W}$ and $\overset{―}{v}$ are the matrix means.

To align responses to the acoustic input, spike latencies at each CF were adjusted for cochlear traveling-wave delay by selecting the lag that maximized the cross-correlation between the wavelet spectrogram and the neurogram in that CF channel.

### Automatic word-recognition neural network

To estimate the perceptual consequences of CND, we trained Deep Neural Networks (DNNs) to classify the model-generated neurograms. Analyses were carried out in Python 3.9 with TensorFlow/Keras 2.16 20,21 [34,35]. Inputs were two-dimensional neurograms whose rows were the 71 CF channels and whose columns were 60,000 post-stimulus time bins. Neurograms were flattened to vectors of length (*m· n*), where *m* is the number of time bins and *n* is the number of CF channels, to match the input format of dense network layers.

Two fully connected architectures were evaluated to probe how decoder capacity influences sensitivity to SR-specific deafferentation. *Neural Network* 1 (NN1) comprised five dense layers (128 nodes each) followed by SoftMax output. *Neural Network* 2 (NN2) comprised sixteen dense layers (32 nodes each) to constrain capacity and encourage reliance on robust features that may better track human performance. Hidden layers used ReLU activation and dropout regularization (ρ = 0.03) followed each hidden layer to mitigate overfitting. The output layer had 25 units (the 25 NU-6 words) with SoftMax activation to yield a probability distribution over all lexical classes. Weights were initialized with Glorot uniform. Models were compiled with Adam (default settings), sparse categorical cross-entropy loss [34], and accuracy as the metric. Training was conducted using a fixed number of epochs with data shuffling prior to each epoch to reduce order-related bias. NN1 was trained for 10 epochs; NN2 for 500 epochs to ensure convergence given its greater capacity.

### Training and evaluation corpus

The corpus comprised 25 NU-6 words time-compressed to 65% and reverberated with a 0.3-s decay. To promote generalization, 20 noisy variants per word were created by adding zero-mean Gaussian noise at SNRs from 11 to 30 dB in 1 dB steps. For testing, nine variants per word were generated at SNRs from −30–10 dB in 5 dB steps. Each waveform was resampled to 100 kHz, RMS-normalized, converted to Pascals, and presented to the auditory-periphery model at 90 dB SPL. A single presentation (no repetition averaging) was used to generate each neurogram. The dataset was split 69%/ 31% into training and held-out test set; the same split was used across conditions to enable paired comparisons.

### Neurogram generation and CND manipulation

Three auditory-nerve population configurations represented different CND severities. *Normal Innervation* included 2 low-, 3 medium-, and 7 high-SR fibers per CF, approximating classic cat distributions [36]. *High-SR only* modeled selective loss of low- and medium-SR fibers while retaining 7 high-SR fibers per CF. *Severe Neural Loss* retained 3 high-SR fibers per CF (~75% ANF loss). OHC function was normal in all conditions. Performance was evaluated on the held-out test set using accuracy and confusion matrices to quantify how word recognition degrades as a function of neural survival pattern.

### Statistical analysis

Analyses were performed in Python using open-source libraries, including Pingouin statistical package [37]. Two-sample independent t-tests compared two-dimensional correlation coefficients across fiber types. Pearson’s r assessed the relationship between age and word recognition scores with 65% time compression and reverberation (WRS_65%_). To evaluate the relative contributions of age, standard audiometric frequencies (PTA_St_), and extended high frequencies (PTA_EHF_) to word recognition score, we fit the multiple linear regression:

WRS_65%_ ~ Age + PTA_St_ + PTA_EHF_

Model outputs were examined to determine the statistical significance and relative contribution (e.g., standardized beta coefficients or relative importance metrics) of each predictor variable. Statistical significance was defined as p < 0.05. Where multiple pairwise tests were conducted, p-values were adjusted using Benjamini-Hochberg false discovery rate procedure. All preprocessing, modeling, and visualization were implemented in reproducible Python workflows.

## Results

**Fig 1:**
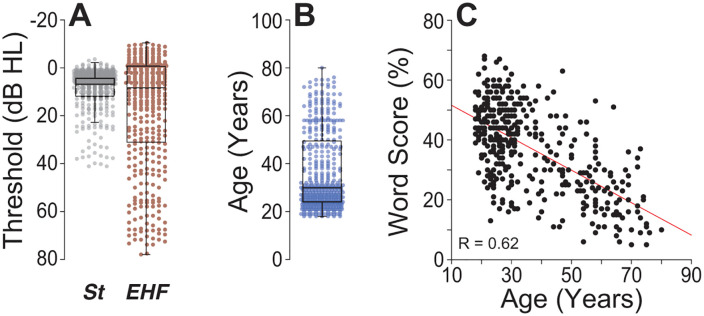
Audiometry and age effects on degraded speech recognition. A: Distribution of mean audiometric thresholds across the study cohort (n = 365), assessed at both standard audiometric frequencies (St) and extended-high-frequencies (EHF). Thresholds were averaged across ears. B: Age distribution of participants, who ranged from 18 to 80 years. C: Relationship between participant age and word recognition performance. Word scores were measured using time-compressed (65%) and reverberant NU-6 words. Each dot represents an individual participant. Pearson correlation coefficients quantify the association between age and speech recognition ability, highlighting a decline in performance with advancing age.

Audiometric thresholds were measured in 395 individuals (220 females, 175 males) aged 18–80 years and cognitively normal (Fig 1A,B). Testing included standard audiometric frequencies (0.25–8 kHz) and extended high frequencies (EHFs; 9–16 kHz). Word-recognition performance was assessed with NU-6 lists presented at suprathreshold levels under degraded conditions (65% time compression, 0.3-s reverberation). Performance declined with age (r = −0.62, p < 0.001) and showed wide inter-individual variability, ranging from ~70% down to <10% correct (Fig 1C). To evaluate how much of this variance was explained by audibility, we fit a multiple linear regression with word score as the dependent variable and age, standard pure-tone average (PTA_St_) and EHF average (PTA_EHF_) as predictors. The model was highly significant (adjusted R^2^ = 0.41, p < 2.04.10^-45^). Poorer PTA_EHF_ was associated with lower scores (p = 0.002), yet age remained an independent predictor (p = 0.001) after accounting for PTA_St_ and PTA_EHF_. Thus, factors beyond threshold sensitivity, but associated with aging, contribute to the decline in word recognition under adverse listening conditions.

**Fig 2:**
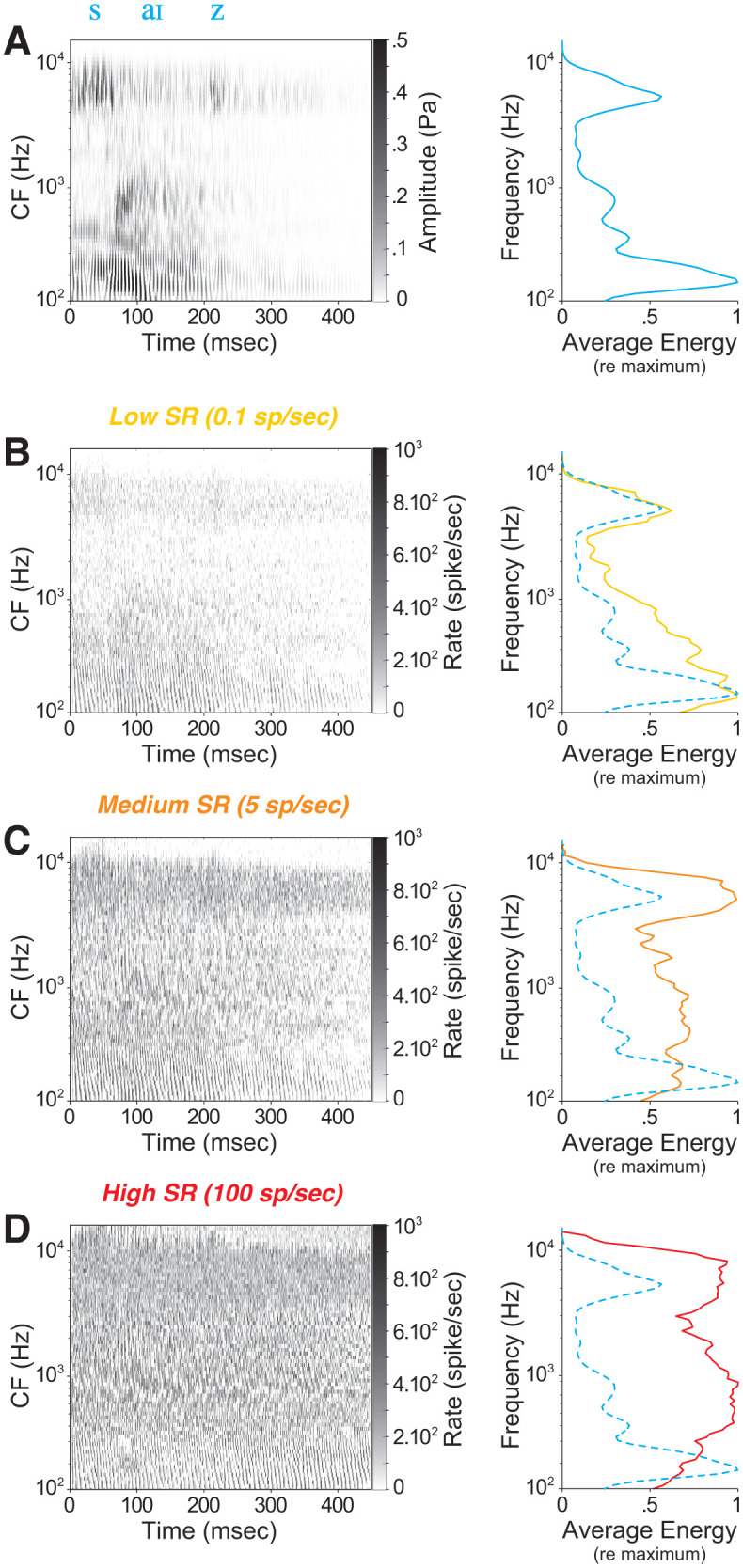
Spectro-temporal stimulus structure and SR-specific ANF responses. Panel A shows the spectrogram of the word “size” (*left*), and the time-averaged energy distribution across frequency bins (*right*), normalized by the maximum value. Panels B through D show the neurograms (left) and rate-place profiles (right) for single low-, medium-, and high-SR ANFs at each CF in response to the same stimulus presented at 90 dB SPL. ANF firing rates from the model output (X axis) are displayed as a function of CF (Y axis). Average responses are computed across time and normalized by the maximum value across CF. For comparison, the average acoustic energy from panel A is superimposed (dashed lines) on each of the rate-place profiles.

To probe a neural basis for these effects, we simulated ANF responses to the same temporally degraded NU-6 stimuli used behaviorally. Given the selective vulnerability of low- and medium-SR fibers to CND [8,9,14], we asked how each SR group contributes to the neural encoding of speech under challenging conditions. Using the phenomenological auditory periphery model of Zilany et al. [32], we computed neurograms across CF for each SR class in response to the word “size” presented at 90 dB SPL (Fig 2B–D). For each SR group, we derived a “rate-place” profile by averaging firing rate over time (Fig 2B–D, right column) and compared it to the stimulus spectral profile from its wavelet spectrogram (Fig 2A). Qualitative comparison indicates that the stimulus structure is better preserved in low-SR, and to a lesser extent medium-SR, neurograms than in high-SR neurograms at this suprathreshold level.

**Fig 3:**
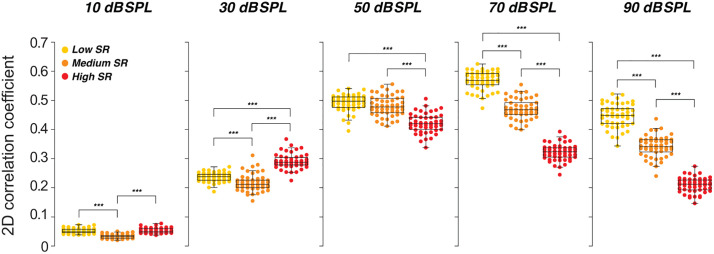
Similarity of acoustic and neural representations across SR groups and levels. Pearson correlations (r) between stimulus spectrogram and corresponding ANF neurogram are plotted for low-, medium-, and high-SR fibers at multiple presentation levels. Each point is one of 50 NU-6 words (65% time-compressed, 0.3-s reverberation). Higher r indicates closer spectro-temporal fidelity. Asterisks mark between-group differences (*** p < 0.001).

We quantified these observations by computing two-dimensional Pearson correlations between each word’s wavelet spectrogram and the corresponding neurograms for each SR group at five levels (10–90 dB SPL). As shown in Fig 3, at low levels (10–30 dB SPL) high-SR fibers produced correlations comparable to, or higher than, those of low- and medium-SR fibers. Above 50 dB SPL, however, spectro-temporal fidelity in high-SR fibers declined sharply, whereas low- and medium-SR fibers maintained significantly higher accuracy. This pattern aligns with known reductions in envelope phase-locking and saturation of high-SR fibers at high SPLs [38]. These results suggest that, at suprathreshold levels, low- and medium-SR fibers disproportionately support the frequency-specific speech cues needed for word recognition in acoustically complex scenes.

**Fig 4:**
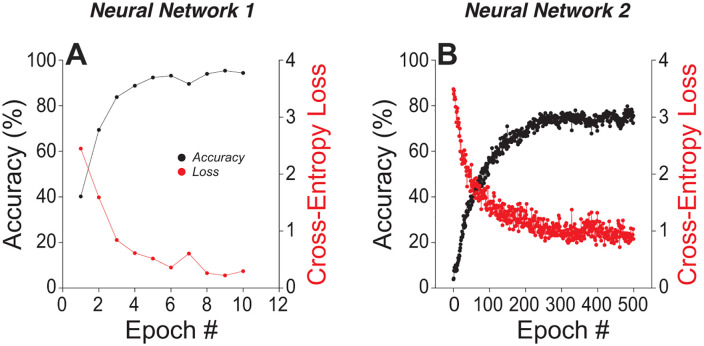
Training performance of neural decoders on simulated ANF neurograms. Learning curves (loss and accuracy) for two fully connected models trained to classify 25 NU-6 words from neurograms. A, NN1: five dense layers (128 units each), ReLU, dropout = 0.03, trained for 10 epochs. B, NN2: sixteen dense layers (32 units each), ReLU, dropout = 0.03, trained for 500 epochs. Both models used the Adam optimizer with sparse categorical cross-entropy. Training inputs were neurograms generated from model responses to speech tokens presented at 11-30 dB SNR in 1 dB steps.

To test whether these encoding differences plausibly impact word-recognition performance, we trained neural networks decoders to classify AN neurograms, providing a simplified readout of central processing. Two architectures were examined: a shallower, wider model (NN1) and a deeper, narrower model (NN2). Both were trained on neurograms evoked by 25 time-compressed, reverberant NU-6 words across multiple SNRs. NN1 converged rapidly, exceeding 90% accuracy within five epochs and remaining near ceiling (Fig 4). NN2 learned more slowly and plateaued below 80% after 500 epochs. The latter aligns more closely with human performance on the same materials, which did not exceed ~70% (Fig 1C). These differences suggests that NN1 likely overfits idiosyncratic regularities that a biological system would likely not exploit, whereas NN2’s constraints yield behavior closer to human limits. We therefore used NN2 as the primary decoder for evaluating the perceptual impact of neural degradation.

**Fig 5:**
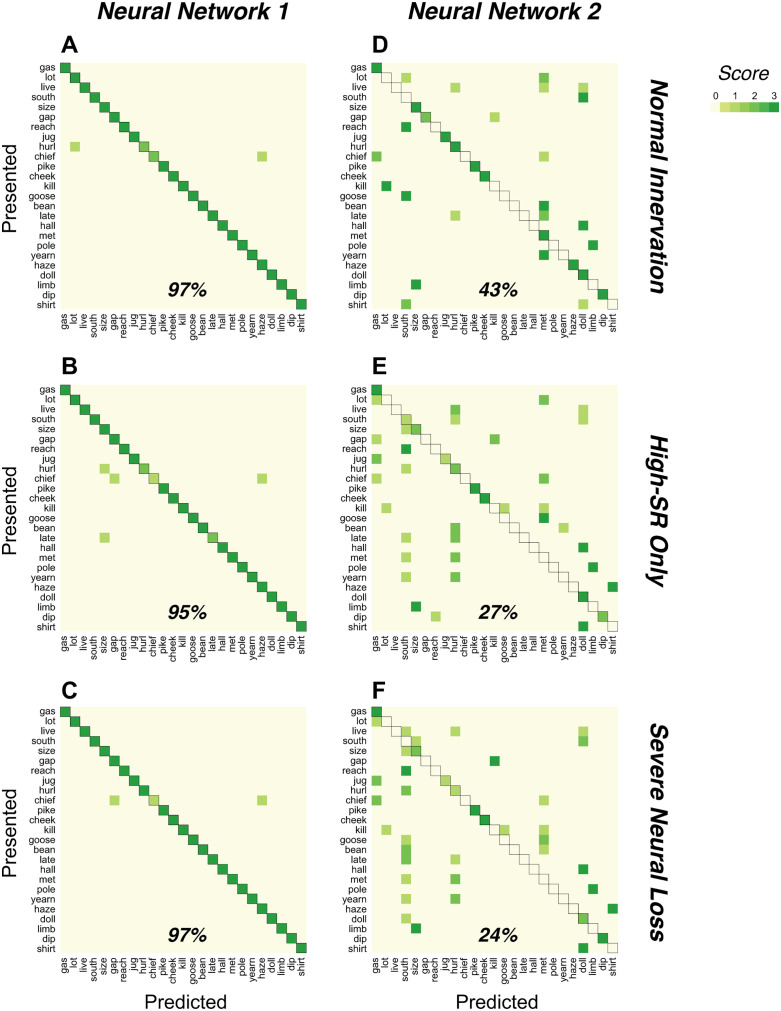
Confusion matrices for word classification across simulated CND. Each matrix summarizes predictions for 25 NU-6 words (65% time-compressed, 0.3-s reverberation) presented at 10, 5 or 0 dB SNR. Neurograms were generated with the Zilany et al. auditory model under three neural conditions: normal innervation, high-SR only, and severe neural loss. A-C: NN1 results for the three conditions. D-F: NN2 results from the same conditions. Y-axes show true word labels; X-axes show predicted labels. Accuracy is indicated by concentration of values along the diagonal; overall percent correct appears below each matrix.

Fig 5 compares NN1 and NN2 across three simulated CND patterns: normal innervation, high-SR only survival, and severe neural loss (see Methods). Neurograms were generated for words presented at 10, 5, and 0 dB SNR.

**Fig 6:**
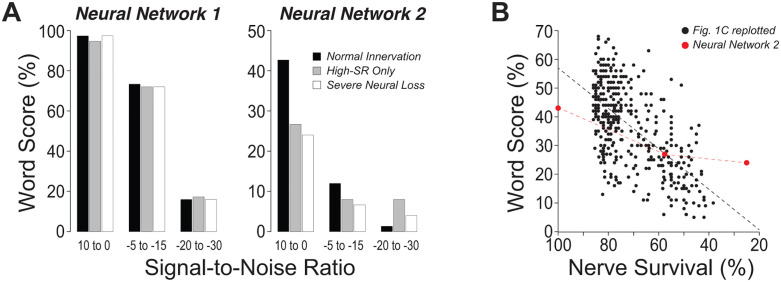
Word-recognition accuracy of the neural decoders across simulated CND and comparison with human data. A: Mean classification accuracy for Neural Network 1 (NN1) and Neural Network 2 (NN2) across three SNR bins (10−0 dB; −5 to -15dB; −20 to -30dB). Curves are shown for three CND profiles generated by the Zilany et al. model: normal innervation (2 low-, 3 mid-, 7 high-SR fibers/ CF), high-SR only (loss of low/mid-SR fibers), and severe neural loss (3 high-SR fibers/CF). B: NN2 accuracy at high SNRs (10−0 dB; red symbols) for the three neural profiles, plotted against a “percent synapse survival” axis. For comparison (gray points), individual word-recognition scores from Fig 1C were mapped to the same axis by converting participant age to predicted auditory-nerve survival using the published age–survival function for normal aging [3].

As summarized in Fig 6A, accuracy decreased with worsening SNR for both decoders, as expected, but only NN2 showed graded declines in performance with increasing neural loss, indicating greater sensitivity to CND. NN2’s terminal accuracy (~ 40% with normal innervation, ~ 20% with loss of low-/medium-SR fibers), better matches the behavioral word-recognition scores obtained from the human cohort, whereas NN1 consistently overestimated word recognition under identical acoustic and neural constraints.

Histopathological data show a strong association between CND and chronological age [3,4], and our behavioral results reveal a similarly robust age dependence of word scores (see Fig 1). Combining these relationships yields an empirical upper bound on the contribution of CND to speech-in-noise performance (Fig 6B). The gap between this bound and model predictions likely reflects additional factors not included here: 1) broadened cochlear tuning and reduced frequency selectivity; 2) residual outer- and inner-hair-cell dysfunction not fully offset by higher presentation levels; and 3) central plasticity and gain changes that come with peripheral de-afferentation [39–42]. Accounting for these factors, along with further decoder optimization, should improve quantitative agreement, as NN2 still under-predicts the full range of age-related deficits, as highlighted in Fig 6B.

## Discussion

This study examined the perceptual consequences of CND, with a focus on the selective loss of low- and medium-SR ANFs that are thought to be critical for encoding speech in challenging acoustic environments [11]. Behaviorally, we confirmed a robust age-related decline in word-recognition among cognitively normal listeners, and regression analyses showed that these deficits could not be fully explained by audiometric thresholds alone, implicating suprathreshold processing impairments. To probe the neural basis of these deficits, we combined a phenomenological model of the auditory periphery with DNN speech decoders trained on spectro-temporal neural representations constrained by human histopathology. Under realistic listening conditions involving time compression, reverberation, and varying noise levels, selective loss of low- and medium-SR fibers produced marked intelligibility declines, consistent with the view that these fibers preserve speech cues in noise.

### Cochlear nerve degeneration and speech-in-noise deficits

A growing body of research supports CND as a contributor to speech-in-noise difficulties, even in individuals with clinically normal audiograms. To date, the only direct evidence linking CND to impaired speech intelligibility comes from histopathological analyses of human temporal bones, which have revealed widespread age-related CND [3,4,13,43] and have associated this neural degeneration with poorer word-recognition performance [43,44], even after accounting for audiometric thresholds [13]. All other supporting evidence remains indirect, relying either on associations between putative biomarkers of CND and suprathreshold perceptual deficits or on studies of clinical populations with conditions presumed to involve CND. In the latter, longitudinal or retrospective investigations have shown disproportionate speech comprehension difficulties in individuals with multiple instances of noise-induced temporary threshold shifts [45], sensorineural hearing loss [25], chronic conductive hearing loss [46], or sudden sensorineural hearing loss [47].

In the former group, several studies have provided physiological evidence consistent with CND-related speech-in-noise deficits that are not explained by elevated audiometric thresholds. As early as 2015, individual differences in envelope-following response (EFR) phase-locking strength and ABR wave I amplitude were correlated with self-reported listening difficulties in noise [48]. Later, electrocochleographic recordings in individuals with normal hearing sensitivity have revealed elevated SP/AP ratios consistent with CND, which were associated with reduced recognition of temporally degraded speech [26,49]. Subsequent work using stimuli optimized to recruit low- and medium-SR fibers showed that EFR magnitude correlated with performance on challenging word recognition tasks [23,50]. Comparable age-related effects have been documented by comparing EFR responses between younger and middle-age adults with normal audiograms, where reduced neural responses predicted greater speech-in-noise deficits and increased listening effort [51].

However, not all investigations reported consistent associations between neural biomarkers and perceptual deficits. In some cases, age-related reductions in ABR wave I growth functions, interpreted as indicative of CND, were not accompanied by measurable speech-in-noise impairments [52]. These inconsistencies may reflect a combination of inter-individual variability in cognitive and attentional factors, limitations of both speech-in-noise and electrophysiological assays to detect subtle impairments, and the complex interplay between peripheral and central auditory processing. Additionally, CND is highly correlated with outer hair cell loss [3,13], so controlling for audiometric threshold automatically reduces the apparent impact of neural loss on perception outcomes.

To better isolate the functional impact of CND, some studies incorporated broader test batteries that include cognitive, psychophysical, electrophysiological, and speech-in-noise measures using stimuli tailored to preferentially recruit low- and medium-SR ANFs. One such study found that neither hearing sensitivity, EFR metrics, nor executive-function measures accounted for the variability in spatial release from speech-on-speech masking [53]. Instead age, a variable highly correlated with CND, emerged as the primary predictor impacting speech-in-noise performance, even after adjusting for audiometric and cognitive factors.

Neuroimaging offers converging support. High-resolution magnetic resonance imaging (MRI) has revealed reduced cross-sectional area and altered microstructural properties of the cochlear nerve in older adults relative to younger controls, with reduced neural synchrony predicting weaker recognition of time-compressed and noise-degraded speech [54]. Magnetoencephalography combined with ABR measures further showed age-related reductions in medial geniculate body activity and cochlear neural onset responses linked to declines in speech-in-noise performance, highlighting combined effects of CND and central auditory pathway dysfunction on speech intelligibility in older adults [55].

### Computational modeling prediction of speech-in-noise deficits

One computational modeling study estimated synapse counts between ANFs and inner hair cells by combining DPOAE and ABR wave I data. Lower predicted counts were associated with greater noise exposure, higher risk of tinnitus, advancing age, and poorer speech-in-noise performance [56]. However, most computational studies have predicted only modest or negligible perceptual consequences of synaptic loss, even under simulations involving dramatic reductions in ANF populations. Early approaches approximated CND by reducing auditory signal fidelity through stochastic undersampling [17,57], which produced greater impairment for brief stimuli (< 20 ms) and steeper threshold vs. signal duration functions in older adults with normal audiograms, but lacked physiological specificity and did not capture SR-specific roles in encoding. Another approach, grounded in signal detection theory, predicted minimal perceptual deficits under extreme deafferentation [18], while assuming equal information across fiber types and limiting performance by ANF variability alone.

More recent models incorporated richer biophysics and decoding stages. A modified Zilany et al. peripheral model [31] paired with a DNN trained to classify digits in noise showed significant intelligibility decline once simulated CND reached 50% [20]. A different auditory model [58] and a sensorineural hearing loss simulator suggested that up to 90% deafferentation might be required to elevate speech recognition thresholds in noise [19]. Another study simulated a large population of ANFs across SR classes and decoded spike patterns into speech features for resynthesis, finding threshold elevation in quiet and in noise with increasing deafferentation, with strongest effects beyond 90% loss [21].

Our work extends these prior efforts in three ways. First, we used ecologically demanding speech materials across multiple SNRs that stress temporal precision and dynamic range, two domains strongly dependent on low- and medium-SR fibers. Second, we simulated ANF responses with the Zilany et al. model [31] while explicitly varied SR-specific survival in line with histopathology. Third, we asked how CND-induced degradation of neurograms impacts decoding by comparing two fully connected NN architectures that differ in depth and width.

Decoder architecture proved pivotal. The shallow and wide network (NN1) quickly achieved high accuracy and was largely insensitive to fiber loss. The deeper, narrower network (NN2) showed CND-driven decrements that paralleled age-related declines in our behavioral cohort. This divergence suggests that over-parameterized decoders can mask biologically meaningful degradations by fitting idiosyncrasies in the degraded input, whereas constrained architectures better approximate human performance limits. We note that inputs were presented at 90 dB SPL to elicit robust suprathreshold responses across SR classes, a choice that likely pushes high-SR fibers toward saturation. This may understate their contribution at everyday levels and should temper direct quantitative comparisons.

### Efferent control and implications for CND modeling

The current simulations omit efferent control. *In vivo*, the medial olivocochlear (MOC) pathway provides dynamic gain regulation at the cochlea, with fast and slow components acting over tens to hundreds of milliseconds [59–62]. These feedback loops can suppress masker-driven basilar-membrane motion, reduce saturation in high-SR fibers, and enhance envelope fidelity when backgrounds fluctuate. MOC drive is also state dependent: it increases with attention and task difficulty, varies across listeners, and can be weakened by aging or neuropathy. Excluding this pathway may therefore either overstate the impact of afferent loss when efferent support would have mitigated masking, or understate resilience in listeners who recruit strong MOC modulation during difficult tasks. Recent subcortical modeling work has begun to formalize these effects, showing that dynamic MOC gain control driven by cochlear-nucleus and inferior-colliculus inputs can account for forward masking and other temporal phenomena relevant to speech coding in noise [63,64]. It must be noted however, that minimally invasive assays of MOC reflex strength consistently show weaker MOC feedback inhibition in human subjects than in commonly used animal models such as guinea pig, cat or mouse [60,65,66], and morphological studies confirm that there is a much sparser MOC innervation in humans than in the well studied animal models [66].

### Limitations and future directions

While our model accounts for a significant portion of the variability observed in human performance, it does not capture the full range of behavioral outcomes. Some of this gap may arise from the peripheral model itself, which captures many nonlinearities but may not reproduce all temporal dynamics of low-SR fibers, including adaptation and masking susceptibilities that are central under degraded conditions. Central compensation is also not modeled. We used monosyllabic words to minimize linguistic context and isolate peripheral coding, but everyday speech provides lexical and semantic redundancy that can mitigate peripheral deficits. Extending tests to sentences and fluctuating maskers will clarify how CND interacts with top-down context. Likewise, our DNN classifiers do not exploit the full range of strategies available to human listeners, which may limit direct numerical alignment.

Another caveat lies in the operational definition of SR classes. Our model adopts SR proportions from classic cat studies. SR distributions are species- and CF-dependent [9,36,67,68]. We also simulated complete loss of low- and medium-SR fibers in some conditions to bracket worst-case scenarios. These assumptions clarify the contributions of each fiber type but may not reflect the graded nature of CND in aging populations.

## Conclusion

Our results support the hypothesis that CND, particularly the selective loss of low- and medium-SR fibers, can meaningfully contribute to the age-related decline in speech recognition under adverse listening conditions. Using spectrally and temporally altered speech, a phenomenological auditory-periphery model, and neural network decoding, we show that SR-specific deafferentation degrades suprathreshold encoding in ways that translate to poorer recognition accuracy. A key insight is that decoder design strongly governs sensitivity to neural loss: over-parameterized networks can obscure biologically meaningful deficits, whereas more constrained architectures yield performance that better tracks human data. These results provide a practical bridge between peripheral neural damage and perceptual outcomes and motivate the use of biologically informed decoders calibrated to human performance ranges. Incorporating efferent feedback, sentence-level context, and human-relevant SR mixtures should refine quantitative predictions and support translation to diagnostics and interventions.
